# Living kidney donation prior to euthanasia: medical, psychological and ethical considerations

**DOI:** 10.3389/ti.2026.16594

**Published:** 2026-07-14

**Authors:** Emma K. Massey, Eline Bunnik, Louise Maasdam, Frank J. M. F. Dor, Jacqueline van de Wetering

**Affiliations:** 1 Section of Nephrology and Transplantation, Department of Internal Medicine, Erasmus MC Transplant Institute, University Medical Center Rotterdam, Rotterdam, Netherlands; 2 Programme of Medical Ethics, History and Philosophy of Medicine, Department of Public Health, Erasmus MC, Rotterdam, Netherlands; 3 Section of HPB and Transplant Surgery, Department of Surgery, Erasmus MC Transplant Institute, University Medical Center Rotterdam, Rotterdam, Netherlands

**Keywords:** bioethics, euthanasia, kidney translant, live donation, psychosocial aspects

## Abstract

Living kidney donation *prior to* euthanasia has never previously been reported and is not incorporated into current clinical guidelines. This study aims to discuss the unique medical, procedural, psychological and ethical considerations of living donation prior to euthanasia based on 3 Dutch cases. Of the 3 candidates, 2 successfully donated without detrimental effects to physical or mental health; one was declined for medical reasons. In two cases, euthanasia has been performed; in one case donation was transformative and the request for euthanasia was withdrawn after donation. Medically, normal life-expectancy after donor nephrectomy should be presumed as euthanasia remains uncertain. Procedurally, timing can create tension between these two processes as postponing euthanasia could be harmful. Psychologically, potential exacerbation in psychopathology should be weighed against the impact of being declined for donation. Ethically, autonomy, capacity to consent and risk-benefit ratio must be rigorously assessed, especially given the degree of suffering in these cases. We conclude that living donation and euthanasia should be seen as independent processes and a euthanasia request should not be considered an absolute contra-indication for living donation. Our initial experiences underscore the need for international dialogue and policy development.

## Graphical Abstract

**Figure:**
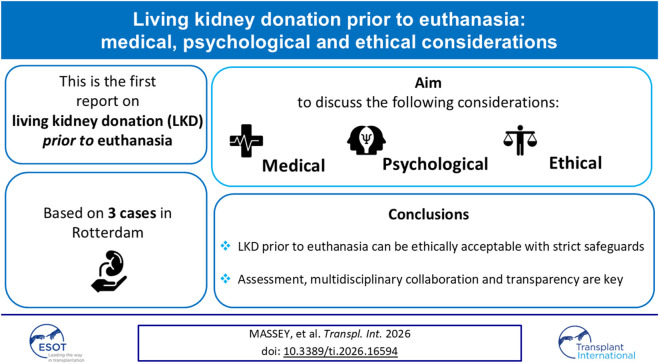

## Introduction

Living donor kidney transplantation affords superior patient survival, graft survival [1] and quality of life [2] compared to other treatments, especially when performed pre-emptively [3]. Transplantation using kidneys from living donors accounted for 39% of kidney transplants globally in 2022 (Global Transplant Observatory). As living donors undergo surgery solely for the benefit of another, minimizing harms is essential. Thorough evaluation processes have been developed to reduce surgical, physical, psychological, and socio-economic harms of donors [4–9]. Family, friends, anonymous donors and even people with terminal illnesses [10] are often highly motivated to donate a kidney. This also applies to people who, in addition to the wish to donate a kidney, also have a wish for euthanasia. Euthanasia is defined as intentionally ending life, at the explicit voluntary and well-considered request of the patient, due to unbearable suffering with no prospect of improvement. Strict due care criteria, according to the Termination of Life on Request and Assisted Suicide (Review Procedures) Act (2002), must be fulfilled by the attending physician (sometimes referred to as Medical Assistance in Dying). Although deceased donation *after* euthanasia has been documented [11, 12] and shown to be have comparable long-term outcomes to controlled donation after circulatory death (DCD-III) and donation after brain death (DBD) [13, 14], living donation *before* euthanasia has never been reported and is absent from clinical guidelines. Evaluation of these individuals forms a new frontier in transplantation. Euthanasia or Medical Assistance in Dying (MAiD) is legal in various countries for patients with medical conditions suffering unbearably without prospect of improvement, including psychiatric illness in some jurisdictions [15]. In the Netherlands, due care criteria must be met (Table 1). Some individuals with psychiatric illnesses requesting euthanasia also express a wish to become a living donor and donate one of their kidneys. Importantly, this study does not incorporate requests for removal of both kidneys or for continuous sedation prior to euthanasia. This article aims to discuss the ethical, psychosocial, and procedural considerations of living donation prior to euthanasia based on three cases from clinical practice. The ultimate aim is to stimulate discussion and policy development.

**TABLE 1:** Due care criteria for euthanasia in the Netherlands.

According to the Dutch Euthanasia Act [Termination of Life on Request and Assisted Suicide (Review Procedures) Act] the physician must
• be satisfied that the patient’s request is voluntary and well considered
• be satisfied that the patient’s suffering is unbearable, with no prospect of improvement
• Have informed the patient about his situation and his prognosis
• Have come to the conclusion, together with the patient, that there is no reasonable alternative in the patient’s situation
• Have consulted at least one other, independent physician, who must see the patient and give a written opinion on whether the due care criteria set out in (1) to (4) have been fulfilled; and
• Have exercised due medical care and attention in terminating the patient’s life or assisting in the patient’s suicide

Source: Is euthanasia allowed in the Netherlands? | Euthanasia | Government.nl.

## Methods

Protocolized medical workup was performed as standard according to the British guideline for living donor kidney transplantation of 2018 [16], adapted to the Dutch situation. According to protocol, medical evaluation included physical examination, imaging, and serology testing. Medical history was obtained from the donor candidate and the General Practitioner. Psychosocial evaluation, conducted by a transplant psychologist using standardized screening tools covered motivation and decision-making, risk and information processing, psychopathology, social resources, and personal resources and ethical considerations [4]. Neither Dutch nor British guidelines list psychiatric illness as an absolute contraindication for living donation, allowing for case-by-case assessment. Candidates were reviewed by a multidisciplinary team including transplant nephrologist, surgeon, anaesthesiologist, nurse practitioner, social worker and psychologist.

Both formal and informal caregivers were approached as sources of information during the assessment process. Standard living donor nephrectomy principles were adhered to in order to maximise donor safety and preserve optimal long-term renal function for the donor. Kidney selection for donation was based on established donor safety principles, whereby the kidney with the less favourable anatomical and/or functional characteristics was preferentially procured, while the donor retained the kidney with the best expected long-term function. This assessment incorporated comparative evaluation of renal parenchyma, split renal function on indication, kidney size, vascular anatomy, and other relevant clinical considerations. For the purposes of this study, donors were given verbal and written information about the aims and procedures. All three gave written informed consent for case review and publication and were given the opportunity to review the case description and manuscript. To ensure privacy and anonymity no names, dates or identifying information were reported. This retrospective file review does not fall under the Medical Research involving Human Subjects Act (WMO) and therefore under Dutch law does not require approval by the Institutional Review Board. All procedures were carried out in accordance with the ethical standards as laid down in the 2024 Revision of the Declaration of Helsinki.

## Results

Three case studies of candidates for living donation prior to euthanasia are presented in Table 2. In all three cases, euthanasia was sought due to suffering from a psychiatric condition. Of the 3 candidates, case 2 was declined for medical reasons, and case 1 and 3 donated. In both cases, donation was successful and no detrimental effects were observed on mental or physical health. In case 1 and 2, euthanasia was performed a minimum of 3 months after donation or conclusion of screening; in case 3 living donation was transformative and the request for euthanasia was subsequently withdrawn. The 3 cases gave rise to unique medical, procedural, psychological and ethical considerations.

**TABLE 2:** Description of donation and euthanasia procedures among three donor candidate**s**.

Case 1 A female aged (20–30 years) who wanted to donate a kidney to a friend with end-stage-kidney disease. This individual’s main motivation to donate was to give her friend a better life expectancy and quality of life. She had been close friends with the recipient for many years. The donor had capacity to make her own decisions. She was well-informed of the procedures and risks and had considered donation over many months. The medical history indicated long-standing mental suffering. She was diagnosed with post-traumatic stress disorder, avoidant personality disorder and chronic depression. She had undergone extensive psychotherapeutic treatments, both in the clinical and outpatient settings to little effect. Various psychotropic medications had been prescribed without effect. Physical evaluation showed no contraindications for kidney donation. Psychological evaluation revealed psychopathology which was elevated when compared to the general population norm scores. She reported a persistent death wish but no acute suidical thoughts or actions at time of work-up. Medication at the time of work-up was an antipsychotic. The donor candidate’s main source of social support was her parents who supported donation. She lived independently in an apartment complex whereby psychological support was available 24 h a day if needed. Her mental healthcare team was consulted during work-up, who indicated that she was considered legally competent and understood the risks and consequences of kidney donation. They supported donation and were available in the post-donation period. It was estimated, in consultation with the candidate, her own psychologist and the transplant psychologist, that mental health would not be negatively influenced by the donation procedure. The candidate expressed that living donation would add meaning to her life. After multidisciplinary consultation, approval was given for kidney donation. Prior to initiation of donation work-up this individual had sought but not received approval for euthanasia due to chronic psychiatric illness. In the Netherlands, regulations stipulate that all reasonable attempts must have been undertaken to treat the psychiatric illness(es). The patient had been obligated to attempt one final treatment option that had recently been introduced to treat chronic depression. This candidate donated a kidney to her intended recipient. The donation operation and the recovery afterwards were uncomplicated. The donor attended the 6 weeks and 3 months check-up in the outpatient clinic. Psychological monitoring by telephone took place in the months after donation. Physical recovery was uncomplicated. There were no discernible self-reported changes in mental health after donation. The individual proceeded with her euthanasia application process which was carried out in >3 months after donation.
Case 2 A female (aged 50–60 years) who wanted to donate a kidney to a first-degree family member with a hereditary kidney condition. The potential donor was motivated to improve the quality of life for the recipient and to avoid loss for other family members. She had capacity and was well-informed about the risks and procedures. She presented the decision as well thought through over time and autonomous. Her medical history included a chronic disease that was not considered a contraindication for donation. Mental health history revealed a diagnosis of post-traumatic stress disorder, dysthymia and (unspecified) personality disorder. She had a history of multiple suicide attempts but there was no evidence for acute suicidal thoughts or actions at the time of work-up. She had previously undergone various psychotherapeutic and psychotropic treatments for her symptoms without success. Psychological evaluation showed elevated psychopathology compared to the general population. She had a stable regime of psychotropic medication. She received psychological support once every 2 weeks and more often on request. Review of the candidate’s records revealed a history of abuse by the potential recipient. Due to this, concerns were raised regarding the ability of the candidate to make a donation decision of her own free will. Her residential and outpatient mental healthcare providers were consulted to gain greater insight into her motivation, current symptoms and treatment. Providers indicated that patient was motivated for donation and could adequately monitor her mental state, cope with symptoms and request support when needed. Both residential and outpatient teams were willing to offer support in the post-donation period and estimated that the individual was sufficiently resilient to undergo the procedure. Additionally in this case, the liaison psychiatrist was consulted regarding mental health stability and the combination of nephrectomy and need for (psychotropic) medication in the future. Prior to initiation of donation work-up this individual had sought but not yet received approval for euthanasia due to chronic psychiatric illness. At the beginning of work-up she had been informed that she had to undergo a new treatment which had been recently introduced in order to satisfy the condition of having tried all potential treatments. Consequently, euthanasia was not likely within the coming 6 months according to her healthcare providers. This requirement was later withdrawn. The physical examinations showed a moderate renal function which is a contraindication for kidney donation. Due to this medical contraindication this candidate was not accepted as a living donor. The decision was communicated and annual follow-up of her blood pressure and renal function has been advised. Psychological support was provided by the transplant psychologist after the decision was communicated. This individual proceeded with her euthanasia process which was carried out in the subsequent year.
Case 3 A female (aged 60–70 years) who wanted to donate a kidney anonymously in order to contribute something to society as one of her final acts before euthanasia. The candidate had capacity and understood the risks of the surgery. Her medical history revealed surgical interventions that were not considered contraindications for living donation. Mental health history revealed a diagnosis of chronic depression and a sleep disorder. The candidate had undergone extensive psychotherapy and psychiatric treatments in the clinical and outpatient setting, as well as psychotropic medication. There had been suicide ideation in the past but no acute suicidal thoughts or actions during work-up. Physical evaluation showed no contraindications for kidney donation. Psychosocial evaluation demonstrated elevated psychopathology compared to the general population. She had maintenance psychotropic medication for these symptoms. She was not undergoing psychotherapy at the time of donor work-up. Social support was provided by her adult children who supported donation. At the time of donation work-up, the candidate had undergone screening for euthanasia and received approval. She had a written statement registered with the General practitioner requesting euthanasia at a point in time when suffering becomes unbearable. Given the chronicity of the psychological symptoms which were stable with maintenance medication and adequate understanding of the process and risks, it was estimated that donation would not negatively influence mental health. The candidate herself agreed with this estimation. After multidisciplinary consultation with the multidisciplinary transplant team, approval was given for kidney donation. The donation operation and the recovery afterwards were uncomplicated. She attended the 6 weeks surgical check-up and 3 months nephrological check-up. Physical recovery was uncomplicated other than a minor, temporary physical complication. Now, 10 years after donation, her kidney function is stable and she is in good physical condition. She visits the psychologist once every 3 weeks for ongoing mental health support. After living kidney donation, the donor retracted her euthanasia request and no longer had an active euthanasia wish. She described living donation as a turning point in her life, after which her mental health improved.

First, as these patients were suffering so severely from psychiatric illnesses, there were concerns about their ability to provide informed consent. All three candidates had to demonstrate capacity to make informed decisions and adequate understanding of the procedures and consequences of donation. Even though all three candidates were convinced at the time of work-up that they would proceed with euthanasia, we asked them to consider a scenario in which they may not undergo euthanasia and would live the rest of their lives with only one kidney to make sure that they understood that this scenario was also possible. In all three cases, there was consensus that candidates’ mental health problems did not cloud their capacity for judgement, risk perception and informed decision-making. Candidates could relay the most important risks and reported realistic expectations of the process of surgery and recovery. As with all potential kidney donors, we also assessed the voluntariness of these donations. In cases 1 and 2 the donor was known to the recipient, the candidates had offered the kidneys themselves, and the work-up did not reveal any suggestion of coercion. Case 3 was an unspecified anonymous (non-directed) donor; therefore, there was no potential for pressure from a potential recipient.

In living donation ethics, it is necessary to balance respect for the autonomy of the candidate with the duty to protect them as healthy individuals against the risks and burdens of living donation. In people who are considering or accepted for euthanasia, and are thus already suffering unbearably, the principle of non-maleficence may apply even more. Moreover, (the timing of) euthanasia is not certain and there is also a chance that the donor candidate may live many years after donation. Therefore, euthanasia was not factored into the medical decision-making: we assumed normal life expectancy due to the uncertainty of euthanasia when assessing medical risk (kidney functioning and potential for short and long term complications). This line of reasoning is supported by case 3 whereby the donor withdrew her euthanasia request after donation, supporting the need for conventional risk thresholds.

With regard to psychological risks and benefits, careful psychosocial evaluation is imperative given that all three cases had a euthanasia wish due to unbearable psychological suffering. There is some evidence that chronic psychopathology may be associated with poorer mental health outcomes of living donation [9, 17]. While all donors had a chronic mental illness, they were stable during evaluation meaning that there were no recent major fluctuations in psychopathology or changes in mental health status. The potential impact on further exacerbation of mental health symptoms, as well as their ability to cope with pain or potential complications, were discussed with all three candidates. Candidates themselves did not expect that donation would have a negative impact on mental health, but rather a positive impact. Candidates believed that donation could give meaning to the final phase of their lives; donation could be a positive event in an otherwise difficult life. For case 3, donation was transformative and led to withdrawal of the euthanasia request. In this case, the psychological benefits of donation clearly outweighed the risks. The risk of psychological harm from donation was weighed against the harm of being declined as a donor. Especially for specified (directed) donations, the impact of being declined may be significant due to the consequences for the intended recipient. Cases 1 and 2 viewed donation refusal as more harmful than the procedure itself. There is evidence from research that being turned down as a donor can result in feelings of guilt toward the recipient, feelings of disappointment, and in some cases anger when the candidate is not in agreement with the decision [18, 19]. At our centre, psychiatric illness is not an absolute contraindication, which facilitates honest disclosure and allows adequate assessment, intervention and support. We made certain that all three candidates received social support through formal channels (mental health professionals, residential support workers) and informal channels (family, friends) and that mental health professionals involved in their care were available to offer treatment if needed post-donation, in addition to the support provided by the transplant psychologist.

In living donation ethics, it is necessary to balance respect for the donor’s autonomy with the duty to protect against potential harms. In the case of living donation prior to euthanasia, harms could be induced by hastening transplantation or having to postpone euthanasia. For the recipient, it is undesirable to undergo transplant earlier than medically necessary. For the donor candidate, having to delay euthanasia to make transplant possible may prolong suffering and may be experienced as coercive. Transparent communication and planning is therefore key. Voucher systems [20], though not currently used in Europe, may help decouple timing in such cases. In cases 1 and 2, directed deceased donation following euthanasia had been considered by the candidates and could have avoided the risks of surgery and burden of recovery. However, directed deceased donation is legally not permitted in the Netherlands (the ethics of which are explored elsewhere [21]). Only through living donation could they direct their organs to their loved ones. Moreover, they preferred to die at home to minimize distress of family members. Living donation prior to euthanasia was at the time the only way to fulfil all of these wishes.

Finally, the wider societal implications must be considered. Deceased organ donation is based on altruism and trust in the transplant system among the general public. It is not clear whether living donation prior to euthanasia, particularly in the context of psychiatric illness, will influence public trust in the transplant system. Therefore, careful and transparent evaluation is critical.

## Discussion

With the rising number of euthanasia requests due to unbearable (psychiatric) suffering in the Netherlands [22], this type of donor candidacy may become more common in the future. Euthanasia is also legally permitted in other countries and under consideration in new legislation elsewhere [23]. As a result, similar requests for living donation prior to euthanasia may arise internationally. To facilitate ethically responsible transplant care, open dialogue, and development of clinical guidance is necessary. Publication of these cases can not only stimulate dialogue but provide a benchmark against which others can be compared [24]. We urge transplant professionals to approach such requests seriously rather than dismissing them outright. As shown here, careful evaluation can lead to successful living donor transplantation without harm to the donor. Although each case began with the same intent, the outcomes differed, providing unique insight into motivations and the emotional and clinical complexities involved. Based on our experiences, we have outlined a number of challenges and lessons learned.

Firstly, decision-making around living donor candidacy must remain independent from the euthanasia request. The medical assessment should presume the donor may live long after donation, as euthanasia may ultimately not proceed. The evaluation must safeguard against any deterioration in physical or mental health, ensuring that withdrawal of the euthanasia request does not expose the donor to new health risks. A multidisciplinary post-donation care plan is recommended. Secondly, core ethical principles of living donation are especially critical in these cases. Rigorous assessment of capacity to give informed consent and risk-benefit ratio are essential. Multidisciplinary collaboration—including specialized mental health professionals, ethicists, and external professionals involved in the candidate’s care—is vital to ensure consensus on candidacy. Specialized knowledge of euthanasia, particularly in psychiatric cases, is often limited within transplant teams; consulting national euthanasia experts or committees may provide necessary support. Thirdly, these cases were emotionally taxing for the transplant team, especially due to the psychiatric suffering and young age of one candidate. Personal beliefs, stigma, and societal taboos around euthanasia may conflict with professional responsibilities. Institutional support structures—such as supervision, peer-support, and debriefings—should be readily accessible. Learning from prior cases, as presented here, may help overcome barriers, and centralized centers of excellence could consolidate expertise. Finally, there is limited understanding of stakeholder perspectives on living donation prior to euthanasia. Qualitative research involving donors and recipients could help clarify motivations, perceived risks, and benefits. One area to be investigated is whether individuals in the process of evaluation for euthanasia should be actively informed about the option of living organ donation. In the case of Organ Donation after Euthanasia an important aspect of policy is that organ donation can only discussed after the euthanasia process has been completed and approved. This is to protect the individual against potential coercion or pressure to proceed with euthanasia for the sake of the (deceased) donation. In the case of living donation prior to euthanasia, requiring completion of the euthanasia process prior to a donation discussion may be less relevant, as highlighted by Case 3, whereby the euthanasia request was withdrawn after living donation. Actively informing individuals with a euthanasia wish about living donation could either be experienced as coercive or as an opportunity to do something meaningful prior to death, however, research is currently lacking. Public perceptions also warrant exploration, including potential objections, societal acceptance, and any impact on willingness for deceased donation. Jansen and Gardiner (2024) caution that ethical challenges can pose barriers to clinical implementation of organ donation in the context of euthanasia. Careful consideration of stakeholder perspectives is necessary to guide development of policy and ethically responsible implementation.

Living kidney donation prior to euthanasia poses complex ethical, medical, psychological and societal challenges. It requires careful assessment, multidisciplinary collaboration, and transparent communication. Our findings support the feasible of such donations under rigorous safeguards and suggest directions for future guidance and research.

## Data Availability

The original contributions presented in the study are included in the article/supplementary material, further inquiries can be directed to the corresponding author.
